# Transcriptome Analysis Revealed Hub Genes Related to Tipburn Resistance in Chinese Cabbage (*Brassica rapa* L. ssp. *pekinensis*)

**DOI:** 10.3390/plants14040527

**Published:** 2025-02-09

**Authors:** Yaning Bi, Wenjing Zhang, Yuxiang Yuan, Jianqi Feng, Peiyun Wang, Cong Ding, Yanyan Zhao, Lin Li, Henan Su, Baoming Tian, Fang Wei, Xiaochun Wei, Xiaowei Zhang

**Affiliations:** 1 Institute of Vegetables, Henan Academy of Agricultural Sciences, Graduate T&R Base of Zhengzhou University, Zhengzhou 450002, China; byn010206@126.com (Y.B.); zhangwj050@163.com (W.Z.); yuxiangyuan126@126.com (Y.Y.); zhaoyanyan9621@163.com (Y.Z.); 15037565062@163.com (L.L.); 18810835083@163.com (H.S.); tianbm@zzu.edu.cn (B.T.); fangwei@zzu.edu.cn (F.W.); 2 School of Agricultural Sciences, Zhengzhou University, Zhengzhou 450001, China; 3 Kaifeng Academy of Agriculture and Forestry Sciences, Institute of Chinese Cabbage, Kaifeng 475000, China; ks6918@163.com (J.F.); fengjq18567@163.com (P.W.); d_cong123@163.com (C.D.)

**Keywords:** tipburn, Chinese cabbage, calcium deficiency, RNA-seq

## Abstract

Tipburn is a physiological disease in Chinese cabbage. In recent years, this disease has become increasingly serious, affecting the quality and economic benefits of Chinese cabbage. However, little is known about the molecular mechanism by which calcium deficiency induces tipburn. Therefore, we performed transcriptome analysis on Y578-2 (tipburn-resistant accession) and Y920-2 (tipburn-susceptible accession) to identify the genes involved in the tipburn defense mechanism in Chinese cabbage. In this study, phenotypic observation showed that Y920-2 began to display symptoms on the 10th day of calcium deficiency treatment. Through weighted gene co-expression network analysis (WGCNA), three gene modules that were highly related to tipburn resistance were identified. Analysis of gene expression regulation in the three modules revealed 13 hub genes related to tipburn resistance, which were involved in the cell wall, photosynthesis, transcription factors, hormones, and the stress response, indicating that these factors play an important role in the tipburn response of Chinese cabbage. These transcriptome data and analysis results provide a basis for the study of the molecular mechanism of calcium deficiency-induced tipburn in Chinese cabbage.

## 1. Introduction

Chinese cabbage (*Brassica rapa* L. ssp. *pekinensis*), belonging to the *Brassica* genus in the Brassicaceae family, is one of the most widely distributed vegetable crops in China [1,2,3]. Chinese cabbage is widely consumed because it has high yield and storage resistance and contains many nutrients, such as vitamins, carotenoids, protein, and crude fiber. Chinese cabbage has important economic benefits and extensive research value [4,5].

In vegetable crops, especially leafy vegetables such as Chinese cabbage, lettuce, and cabbage, a decrease in Ca^2+^ below the critical level in rapidly growing tissues leads to physiological disorders and necrosis at developing leaf tips, which is called tipburn [6,7]. Tipburn is generally believed to be a physiological disease caused by a lack of calcium [8,9,10,11]. The incidence of tipburn in Chinese cabbage is usually 10–20% and can be up to 80% in severe cases [12]. Tipburn always develops with pathogen infection, further reducing the yield and quality of Chinese cabbage [13].

Calcium, as an essential mineral element, plays a very important role in plant growth and development [14]. Calcium not only maintains plant cell wall integrity and cell membrane stability, acting as a cell structure component, but also plays an important role in maintaining the balance of physiological activities and improving adaptability to stress as a signal molecule [15]. Ca^2+^ homeostasis in the plant cytoplasm undergoes significant changes in response to various stress signals [16,17]. Calcium deficiency leads to the destruction of the cell wall structure, especially the vessels in phloem and xylem, which hinders the transport and distribution of nutrients and water in plants, thus causing the absorption of nutrients and water to be blocked; this interferes with the normal metabolism of leaves, inhibiting their growth and development, and hindering the leaves from exercising a series of functions such as photosynthesis, ultimately leading to their water loss, atrophy and dryness [18,19].

There is a need to study the molecular mechanism of tipburn in Chinese cabbage. Candidate gene calreticulin 2 (*BrCRT2*) has been mapped using genome-wide association study analysis of 194 tipburn-resistant and -susceptible materials [20]. Sequence variation in susceptible materials led to a decrease in the Ca^2+^-binding ability of the protein, thereby reducing the ability of plants to cope with calcium deficiency stress and ultimately leading to tipburn.

To explore the role of circular RNAs in the process of calcium deficiency-induced tipburn, Wang et al. [21] performed genome-wide analysis of circRNAs in Chinese cabbage under normal and calcium deficiency treatments. The identified differentially expressed circRNAs were involved in stimulus responses, electron carrier activity, cell wall metabolism, plant hormone signal transduction, and transcription factors. In addition, abscisic acid-responsive element binding factor (*ABF2*), a positive regulator of abiotic stress response in the abscisic acid (ABA) pathway, was identified, which may play a role in the response to calcium deficiency stress through the circRNA regulatory pathway. Yuan et al. [22] identified 23 genes related to tipburn through transcriptome analysis of resistant and susceptible materials, and they encoded three protein types, namely calmodulin, Rboh, and CDPK. These genes may promote cell integrity through the Ca^2+^-CaM/CML-CDPK signaling pathway, thereby improving tipburn resistance in Chinese cabbage. Zhang et al. [23] comprehensively analyzed transcriptomic data from control and calcium treatments to determine the molecular mechanism of tipburn. Some differentially expressed genes (DEGs) involved in plant hormone signal transduction, cell wall development, light responses, and stress responses were identified. These DEGs were analyzed using co-expression network analysis to screen the hub genes, providing important evidence for mining the genes related to tipburn caused by calcium deficiency.

Compared with other traits, there are few studies on tipburn in Chinese cabbage and the mechanism regulating tipburn is still unclear, which limits disease resistance breeding in Chinese cabbage. Therefore, the identification of key genes in response to tipburn and analysis of their molecular regulatory mechanisms will help improve tipburn resistance. In this study, Y578-2 (tipburn-resistant material) and Y920-2 (tipburn-susceptible material) were selected for phenotypic investigation, and further transcriptome analysis was performed at three stages after a calcium deficiency treatment and normal calcium treatment. We analyzed these data and mined the genes related to tipburn resistance in Chinese cabbage to understand its molecular mechanism.

## 2. Results

### 2.1. Phenotypic Observations of Tipburn After Calcium Deficiency Treatment

**Figure 1:**
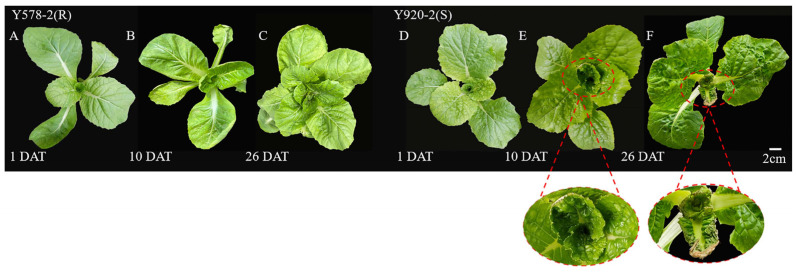
Phenotypes of Chinese cabbage Y578-2 and Y920-2 after calcium deficiency treatment. (**A**–**C**) Phenotype of resistant material Y578-2 at 1, 10, and 26 DAT. (**D**–**F**) Phenotype of susceptible material Y920-2 at 1, 10, and 26 DAT.

Two doubled haploid (DH) lines, tipburn-resistant Y578-2 and -susceptible Y920-2, were screened after repeated experiments in the early stage. In this study, two lines were treated with calcium deficiency. The leaves of Y920-2 exhibited tipburn symptoms at 10 days after calcium deficiency treatment (DAT), and the edge of the inner leaves was dry and papery. At 26 DAT, the disease became increasingly serious, and more than two leaves showed symptoms (Figure 1D–F). However, the leaves of the resistant material Y578-2 did not undergo these changes (Figure 1A–C).

### 2.2. Antioxidant Enzymes Activities of Plant Leaves After Calcium Deficiency Treatment

**Figure 2:**
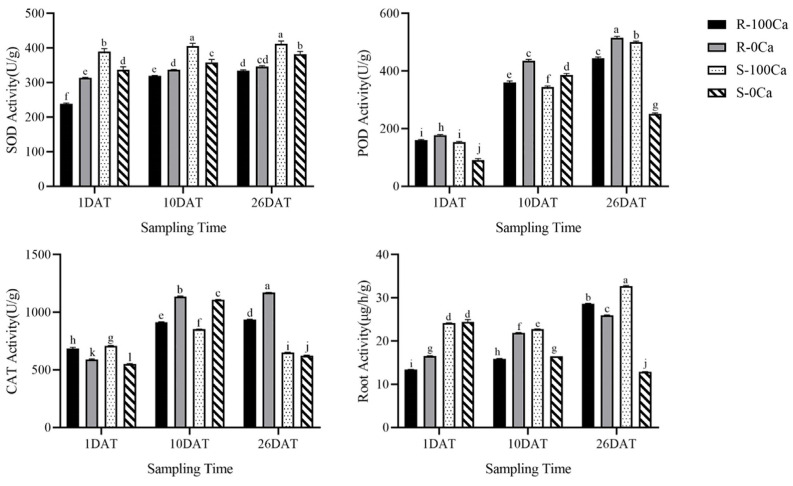
The SOD, POD, CAT and root activity of cultivars cultured with different calcium treatments for different treatment times. The line of each bar represents the standard deviation. The difference between the measured values of different treatments was analyzed (*p* = 0.05). Lowercase letters represent significant differences between different groups.

From the results shown Figure 2, the enzyme activities of superoxide dismutase (SOD), peroxidase (POD), catalase (CAT) in leaves and root activity were changed by calcium concentration and treatment time. In the resistant material Y578-2, the three enzyme activities showed an upward trend with the decrease of calcium concentration and the prolongation of treatment time. However, in the susceptible material Y920-2, the enzyme activities of POD and CAT increased first and then decreased with time after calcium deficiency treatment, and the maximum value appeared at 10 DAT. After calcium deficiency treatment, the root activity of the susceptible material decreased with the prolongation of treatment time, while the other treatments showed an upward trend. Moreover, the root activity of the susceptible material on the 10th and 26th day after calcium deficiency treatment was significantly lower than that for the control calcium concentration.

### 2.3. Sequencing Data Quality Assessment

A total of 36 cDNA libraries were constructed, and the sequencing data are shown in Supplementary Table S1. From the RNA-seq library, approximately 8836 million clean reads were generated, and the Q30 of the clean reads was more than 91.30% in each library, indicating that the clean reads obtained in this study were of high quality. The mapping rate between the samples and the *B. rapa* reference genome (V3.5) ranged from 85.26 to 92.98%. Considering the quality of the data obtained using RNA-seq, it was reliable and met the requirements for subsequent analysis.

A total of 26,267 genes were detected from 36 resistant and susceptible Chinese cabbage samples (Supplementary Table S2). The fragments per kilobase of exon model per million mapped fragments (FPKM) values were analyzed using principal component analysis to determine the consistency of biological repetition (Supplementary Figure S1). Three points of the same color represented three biological replicates from the same sample, and significant differences were observed between resistant and susceptible materials. There were significant differences between the calcium deficiency treatments and the corresponding normal calcium treatments. The expression patterns of these genes were affected by the calcium deficiency treatment and different Chinese cabbage materials.

### 2.4. Statistics of Differentially Expressed Genes

**Figure 3:**
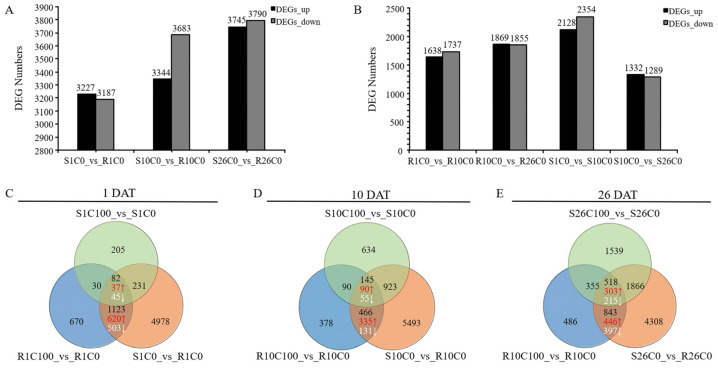
Statistical analysis of genes identified using transcriptome analysis. (**A**,**B**) Number of upregulated and downregulated differentially expressed genes between different comparison groups. (**C**–**E**) Venn diagrams of DEGs at 1, 10, and 26 DAT, respectively. The red numbers indicate the number of genes upregulated by the tipburn-resistant material relative to the tipburn-susceptible material; the white numbers indicate the number of downregulated genes.

Based on the screening threshold of |log_2_ (Fold change)| ≥ 1 and q < 0.05, a total of 6414, 7027, and 7535 DEGs were obtained from the resistant material Y578-2 and susceptible material Y920-2 at 1, 10, and 26 DAT, respectively (Supplementary Table S3). After calcium deficiency treatment, the number of DEGs between resistant and susceptible materials increased with time, and most DEGs were identified at 26 DAT. The number of downregulated genes in the resistant material was greater at 10 DAT compared with the susceptible material, indicating that the response mechanism of the susceptible material was strong at 10 DAT (Figure 3A).

The different periods after the calcium deficiency treatment were compared between resistant and susceptible materials. A total of 3375, 3724, 4482, and 2621 DEGs were identified in R1C0 vs. R10C0, R10C0 vs. R26C0, S1C0 vs. S10C0, and S10C0 vs. S26C0, respectively (Supplementary Table S3). In the susceptible material, most DEGs were identified at 10 DAT compared with the previous period, indicating that the susceptible material had a strong defense response to calcium deficiency stress at 10 DAT and that this period was key for Chinese cabbage to resist tipburn (Figure 3B).

To more accurately screen out resistance-related genes in the resistant material, we conducted joint analysis of the DEGs between resistant and susceptible materials after calcium deficiency treatment and the DEGs of resistant and susceptible materials treated with different calcium concentrations; three groups of DEGs were obtained (Figure 3C–E). These DEGs were identified not only by comparing resistant and susceptible materials but also by comparing calcium concentration treatments of the resistant or susceptible material. Furthermore, the expression levels of these DEGs were significantly higher in resistant material Y578-2 than in susceptible material Y920-2. The three groups of DEGs contained 657, 425, and 749 genes (red numbers in Figure 3), and 1426 DEGs were obtained after merging.

### 2.5. Weighted Gene Co-Expression Network Analysis of DEGs

**Figure 4:**
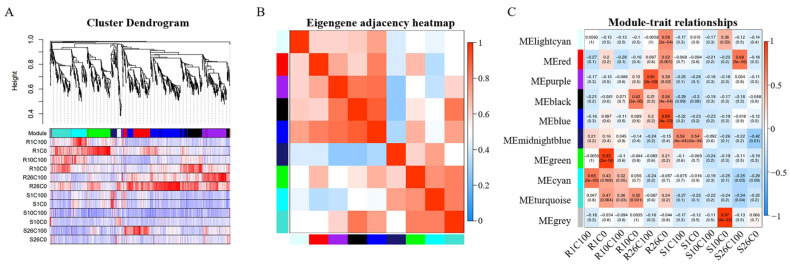
Weighted gene co-expression network analysis (WGCNA). (**A**) Co-expression module division. Visualization of gene modules; the same color indicates that these genes correspond to the same module. (**B**) Module correlation heat map. (**C**) Correlation analysis between modules and traits. Red represents a positive correlation; blue represents a negative correlation. The numbers in each box represent the correlation coefficient (top) and standard error (bottom).

The screened DEGs were subjected to WGCNA, and 1426 genes were assigned to 10 gene expression modules based on the expression pattern. Each module was labeled with different colors, where each branch represented a module, and each line in the branch represented a gene (Figure 4A). The correlation between each module is shown in Figure 4B. Among these modules, the blue and black modules were highly correlated, and the gene expression patterns in these modules were similar.

There was a correlation between these 10 modules and the periods after calcium treatment of resistant and susceptible materials (manifested as different disease resistance levels). These 10 modules were light cyan, red, purple, black, blue, midnight blue, green, cyan, turquoise, and grey (Figure 4C), which contained 32, 159, 191, 165, 308, 47, 174, 119, 146, and 2 genes, respectively. The genes in the grey module did not show an expression pattern and were not analyzed.

The black and blue modules were highly correlated with the resistant material at 26 DAT (R26C0). The black module also had a high correlation with the resistant material at 10 DAT (R10C0), which was consistent with the phenotypic observations. Ten days after calcium deficiency was an important period for resistant material Y578-2 to resist calcium stress. The turquoise module was negatively correlated with the susceptible material Y920-2 at all times after calcium deficiency treatment but positively correlated with the resistant material Y578-2, indicating that the turquoise module was closely related to tipburn resistance in the resistant material. In summary, we speculated that the genes in these three modules strongly responded to calcium deficiency stress resistance in Chinese cabbage and contained important disease resistance genes.

### 2.6. Analysis of the Gene Expression Regulation of Key Modules

**Figure 5:**
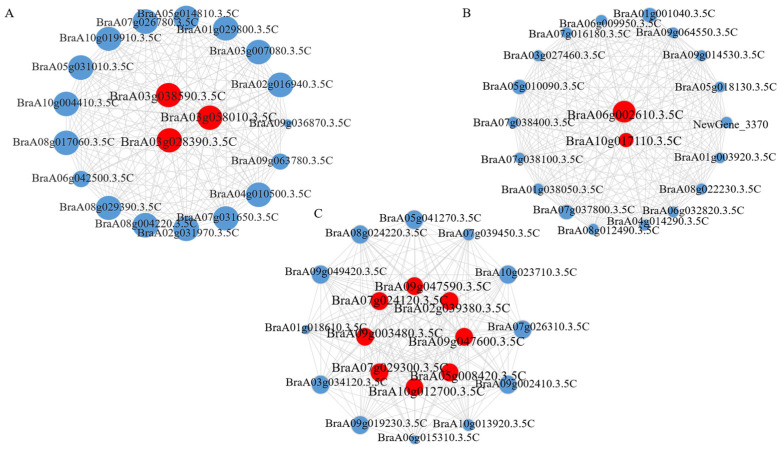
Co-expression network analysis of three key modules. (**A**) The correlation networks in the black module. (**B**) The correlation networks in the blue module. (**C**) The correlation networks in the turquoise module. Candidate hub genes are shown in red.

The expression patterns of the genes in the black, blue, and turquoise modules were visualized by the expression heat map of all genes in the module (Supplementary Figure S2). Some genes with high connectivity in the three modules were subjected to network visualization analysis using Cytoscape 3.8.0 (Figure 5). Combined with the reference documents, three, two, and eight hub genes were identified in the three modules (Table 1). According to the expression trend, three hub genes in the black module, *BraA03g038590.3.5C*, *BraA03g058010.3.5C*, and *BraA03g028390.3.5C*, were highly expressed at 10 and 26 DAT. Hub genes *BraA06g002610.3.5C* and *BraA10g017110.3.5C* in the blue module were upregulated at 26 DAT. The expression levels of eight hub genes in the turquoise module, namely *BraA05g008420.3.5C*, *BraA09g003480.3.5C*, *BraA07g024120.3.5C*, *BraA09g047600.3.5C*, *BraA10g012700.3.5C*, *BraA07g029300.3.5C*, *BraA09g047590.3.5C*, and *BraA02g039380.3.5C*, were upregulated during calcium deficiency treatment in the resistant material Y578-2. The expression levels were significantly higher in the resistant material than in the susceptible material. These 13 hub genes were the key genes.

### 2.7. DEGs in Response to the Tipburn Process

#### 2.7.1. Calcium-Related Genes May Play an Important Role in Tipburn Resistance

**Figure 6:**
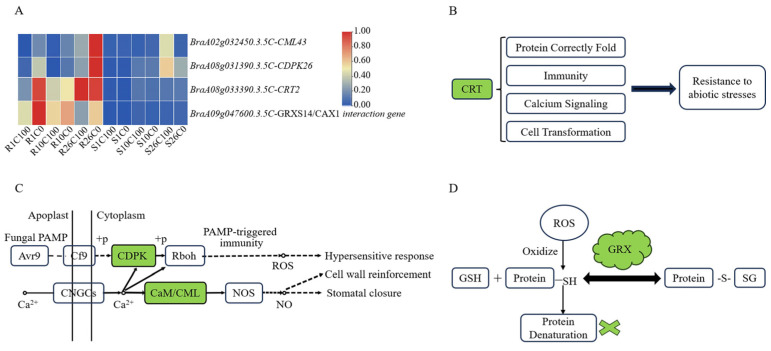
Analysis of calcium-related genes. (**A**) Heat map of calcium-related genes in response to tipburn. (**B**–**D**) Mechanism of action of genes *CRT2*, *CML43*, *CDPK26*, and *GRXS14*.

In this study, four calcium-related genes were screened from three important modules: *CML43*, *CDPK26*, *CRT2*, and *GRXS14* (Figure 6). *CML43* and *CDPK26* were involved in hypersensitive responses, cell wall reinforcement, and stomatal closure, according to the Kyoto Encyclopedia of Genes and Genomes (KEGG) enrichment map. According to gene annotation and references, *CRT2* was involved in the correct folding of proteins in the endoplasmic reticulum, and *GRXS14* was involved in the plant stress response and interacted with the *CAX1* gene.

Among the genes in the aforementioned modules, *BraA02g032450.3.5C* (*CML43*) was significantly upregulated in resistant material Y578-2 at all times after calcium deficiency treatment compared with the control. The expression of *BraA08g031390.3.5C* (*CDPK26*) and *BraA09g047600.3.5C* (*CAX1* interaction gene) was significantly upregulated at 26 DAT compared with the normal calcium treatment. Moreover, *BraA09g047600.3.5C* (*CAX1* interaction gene) and *BraA08g033390.3.5C* (*CRT2*) showed lower expression in the susceptible material and higher expression in the resistant material. This suggests that these four calcium-related genes may play an important role in tipburn resistance in Chinese cabbage.

#### 2.7.2. Stress-Related Genes May Play an Important Role in Calcium Deficiency Resistance

**Figure 7:**
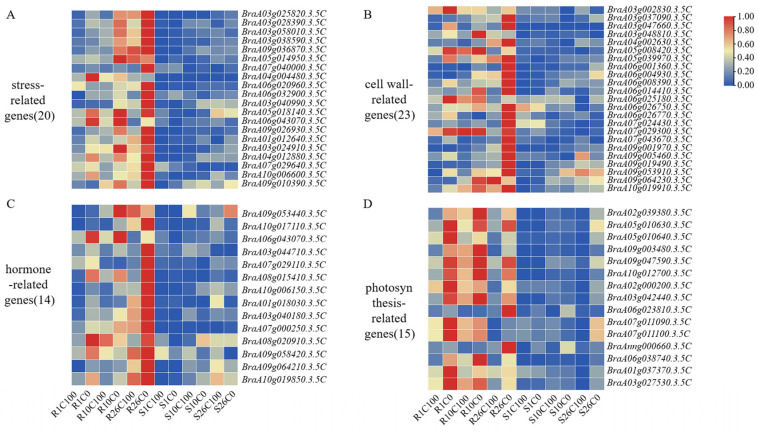
Heat map of differentially expressed genes (DEGs) in response to tipburn. (**A**) Heat map of stress-related genes in response to tipburn. (**B**) Heat map of cell wall-related genes in response to tipburn. (**C**) Heat map of hormone-related genes in response to tipburn. (**D**) Heat map of photosynthesis-related genes in response to tipburn.

According to the analysis, we screened 20 stress-related genes from 3 important modules (Figure 7A). These genes included glutathione peroxidase, *HSP20* family genes, and glutathione S-transferase. These genes were significantly upregulated at 10 and 26 DAT in resistant material Y578-2.

#### 2.7.3. DEGs Related to Cell Walls and Hormones May Play an Important Role in the Late Stage of Calcium Deficiency Resistance

Twenty-three genes related to the cell wall and fourteen genes related to hormones were screened from three important modules (Figure 7B,C), which were significantly upregulated in the resistant material at 26 DAT, with almost no expression or no differential expression in the susceptible material. This indicates that these cell wall- and hormone-related genes play an important role in the late stage of calcium deficiency resistance in Chinese cabbage.

#### 2.7.4. DEGs Associated with Photosynthesis Respond to Calcium Deficiency Stress

The 15 photosynthesis-related genes, including light-harvesting complex II chlorophyll a/b-binding protein, ATP synthase, and cytochrome genes, were hardly expressed or expressed at a low level during calcium deficiency treatment in susceptible material Y920-2 (Figure 7D). In the resistant material, expression was upregulated at all three stages after calcium deficiency treatment compared with the normal calcium control, indicating that photosynthesis-related genes responded to calcium deficiency stress in resistant material Y578-2.

#### 2.7.5. Transcription Factors Showed Different Expression Levels Between Treatments

**Figure 8:**
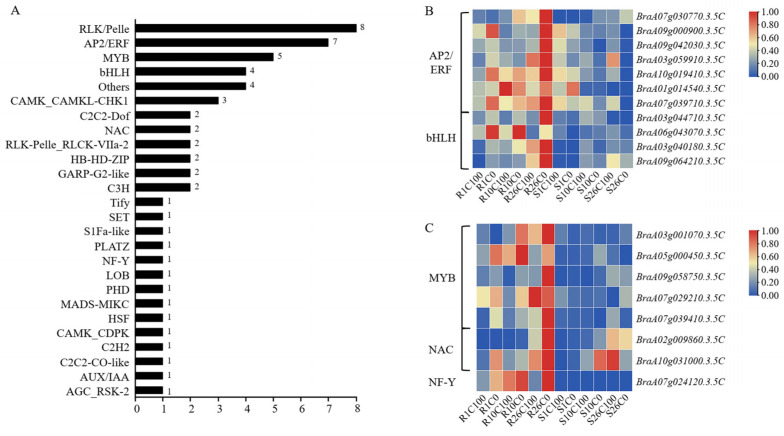
Analysis of transcription factors. (**A**) Number of associated transcription factor families. (**B**) Heat map of AP2/ERF and bHLH transcription factor family gene expression. (**C**) Heat map of MYB, NAC, and NF-Y transcription factor family gene expression.

In plants, many transcription factors play an important role in the abiotic stress response. To determine whether transcription factors play a role in the response to calcium deficiency stress in Chinese cabbage, we analyzed the transcription factors in the three main modules. We screened 57 related genes from 26 transcription factor families, including RLK/Pelle, AP2/ERF, MYB, bHLH, CAMK_CAMKL-CHK1, C2C2-Dof, NAC, and NF-Y (in descending order according to the number of genes they contained) (Figure 8A). We mainly analyzed the AP2/ERF, bHLH, MYB, NAC, and NF-Y transcription factor families, which added up to 19 genes (Figure 8B,C).

In the AP2/ERF family genes, under the calcium deficiency treatment, *BraA07g030770.3.5C* was significantly upregulated at 10 DAT, and *BraA09g000900.3.5C* and *BraA09g042030.3.5C* were significantly upregulated in the resistant material at 26 DAT. The expression levels of the other four genes were significantly higher in the resistant material than in the susceptible material. In the bHLH family genes, *BraA03g044710.3.5C* and *BraA06g043070.3.5C* were significantly upregulated at 26 DAT compared with the control. *BraA03g040180.3.5C* and *BraA09g064210.3.5C* were significantly upregulated in resistant material Y578-2 compared with susceptible material Y920-2.

Of the MYB family genes, *BraA07g029210.3.5C* was differentially expressed between the resistant and susceptible materials at three stages after calcium deficiency treatment. The other four genes were upregulated after the calcium deficiency treatment compared with the normal calcium treatment in resistant material Y578-2. *BraA02g009860.3.5C*, a NAC family gene, was significantly upregulated in all periods after the calcium deficiency treatment compared with the control treatment. *BraA10g031000.3.5C* was differentially expressed between resistant and susceptible materials at 26 DAT. NF-Y family gene *BraA07g024120.3.5C* was significantly upregulated in the resistant material at 26 DAT compared with the normal calcium treatment, and it showed differential expression between resistant and susceptible materials.

Most of the genes in all other families of transcription factors showed diverse expression levels between calcium treatments and between different materials (Supplementary Figure S3).

### 2.8. Verification of RNA-seq Accuracy Using qRT-PCR

**Figure 9:**
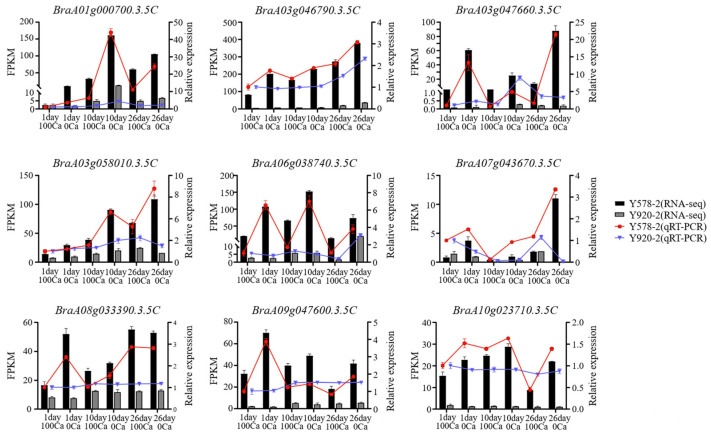
RNA-seq and qRT-PCR consistency checks. Black and gray bars represent the transcriptomic data of resistant and susceptible materials, respectively. The red and blue bars represent the qRT-PCR data of resistant and susceptible materials, respectively. The line of each bar represents standard deviation.

To verify the accuracy of the RNA-seq data, we randomly selected nine DEGs, comprising two calcium-related genes (*BraA08g033390.3.5C* and *BraA09g047600.3.5C*), one gene involved in stress response (*BraA03g058010.3.5C*), two cell wall-related genes (*BraA03g047660.3.5C* and *BraA07g043670.3.5C*), one gene related to photosynthesis (*BraA06g038740.3.5C*), one gene from the black module (*BraA01g000700.3.5C*), one gene from the blue module (*BraA03g046790.3.5C*), and one gene from the turquoise module (*BraA10g023710.3.5C*), for qRT-PCR verification. For each gene, we performed a one-way ANOVA test on the transcriptome FPKM values and analyzed qPCR data of the two materials, and the results are listed in Supplementary Table S5. qRT-PCR showed that the gene expression changes were basically consistent with those observed in RNA-seq (Figure 9), indicating that the reliability of transcriptome data was high.

## 3. Discussion

Tipburn is a common disease in Chinese cabbage production, mainly occurring on young leaves, which causes the cabbage to lose its edible and commodity values and reduces its yield. However, the molecular regulation mechanism of Chinese cabbage tipburn is still unclear. In this study, Y578-2, a tipburn-resistant material, did not show tipburn symptoms after calcium deficiency treatment. After calcium deficiency treatment, the margin of the inner leaves became dry and yellow, and the mesophyll was dry and paper-like in Chinese cabbage variety Y920-2, a tipburn-susceptible material. The characterized morphology of these materials makes them ideal candidates for studying the molecular regulation mechanism of tipburn in Chinese cabbage, making the subsequent analysis data and results more reliable.

Tipburn is a physiological disease caused by calcium deficiency. The intracellular Ca^2+^ level is strictly controlled by the Ca^2+^ storage and transport system, thereby regulating the cellular response to various types of environmental stimulation [24]. The most typical Ca^2+^ transporters are ACA (P_2B_-type Ca^2+^-ATPase pump), CAX (Ca^2+^/H^+^ antiporter) located in vacuoles, and ECA (P_2A_-type Ca^2+^-ATPase pump) proteins located in endoplasmic reticulum, which catalyze Ca^2+^ influx or efflux to regulate the change in the cytoplasmic Ca^2+^ concentration [25,26,27]. In this study, we screened four calcium-related genes from three important modules, of which *BraA09g047600.3.5C* was a hub gene analyzed using WGCNA, encoding the GRXS14 protein and interacting with CAX1 at the same time. It was upregulated in the resistant material after calcium deficiency treatment and significantly different in resistant and susceptible materials. Its homologous gene *AT3G54900* was found to inhibit the sensitivity of yeast cells to H_2_O_2_ and protein oxidation, and it played a role in early seedling growth under H_2_O_2_ stress [28]. The *LeGRXS14* gene in tomato is related to salt stress resistance [29], and *OsGRX20* enhances the tolerance of rice to multiple stressors [30]. *BraA09g047600.3.5C* may also be involved in tipburn resistance in Chinese cabbage by regulating the absorption and transport of calcium ions.

The plant cell wall is a cell structure composed of structural proteins and polysaccharides such as cellulose, hemicellulose, and pectin. In addition to being the main structural component of the cell, the cell wall undertakes the functions of mechanical support, maintenance, and determination of cell morphology. It also plays an important role in plant growth and development [31] and resistance to various biotic and abiotic stressors [32,33], and it affects the agronomic traits of crops [34]. Xyloglucan is the most abundant hemicellulose component in the primary wall of dicotyledonous plants, accounting for approximately 20–25% of the cell wall weight [35]. Plants are often subjected to various biotic and abiotic stressors throughout their life cycle. Consequently, plants adopt a variety of defense strategies to cope with adversity, such as changing the structure and composition of their cell walls, expressing stress-resistant genes, including genes related to xyloglucan metabolism [36,37,38,39,40], and activating resistance-related signals [41]. Hub genes *BraA07g029300.3.5C* and *BraA05g008420.3.5C* analyzed using WGCNA were shown to encode xyloglucan transferase, which belongs to a class of enzymes related to xyloglucan metabolism, and they were significantly differentially expressed between resistant and susceptible materials. Therefore, we speculated that *BraA07g029300.3.5C* and *BraA05g008420.3.5C* play roles in tipburn resistance by regulating cell wall metabolism.

Transcription factors play key roles in the initiation and regulation of gene transcription [42]. Among these transcription factors, the NF-Y and LRR-RLK transcription factor families play important roles in the plant stress response and plant growth and development [43,44]. Previous studies have shown that the *CALRR1* gene in pepper can be induced by salt, abscisic acid, and other stressors [45]. Xu et al. [46] isolated *NtLRR1* and *NtLRR2* genes in tobacco and found that they were involved in the salt stress response of tobacco. It was found that *AT1G51620* played an important role in the ABA signal and various stress responses [47]. We screened one NF-Y and three LRR-RLK family genes, among which *BraA07g024120.3.5C* and *BraA06g002610.3.5C* were hub genes analyzed using WGCNA and were significantly upregulated after calcium deficiency treatment in the resistant material but basically not expressed in the susceptible material. Therefore, *BraA07g024120.3.5C* and *BraA06g002610.3.5C* may be involved in the resistance of Chinese cabbage to calcium deficiency stress by enhancing or inhibiting the transcription of other genes.

As a key endogenous factor mediating the plant stress response, plant hormones are the integration center of plant responses to environmental stimuli, play an important role in the plant defense response, and achieve fine regulation of the stress response through complex signal networks between different hormone signaling pathways [48,49,50]. ABA is an important signal molecule for plant responses to abiotic stress. When plants are subjected to abiotic stress, such as drought and salt stress, they rapidly accumulate ABA, thereby activating the stress resistance response; when the environment is improved, ABA is reduced to the basic level, which is conducive to plant growth [51,52]. A previous study found that ABA accumulated during Ca^2+^ deficiency stress, indicating that ABA plays a regulatory role in response to Ca^2+^ deficiency-induced tipburn in *Brassica rapa* [21]. In this study, hub gene *BraA10g017110.3.5C*, analyzed using WGCNA, was involved in the abscisic acid signaling pathway, which was upregulated after calcium deficiency treatment in the resistant material but was not differentially expressed in the susceptible material. It was significantly differentially expressed between resistant and susceptible materials. Therefore, *BraA10g017110.3.5C* may play a role in tipburn resistance.

Light-harvesting chlorophyll a/b-binding protein (Lhc) is involved in photosynthesis. In advanced plants, *Lhc* family genes regulate photosynthesis, which plays an important role in the response to various stressors and in maintaining plant survival [53,54,55,56]. Han et al. [57] showed that many *Lhc* genes play an important role in the regulation of plant stress response and plant resistance. To clarify the response of *Lhc* genes to stress, Zhao et al. [58] analyzed the expression of *Lhc* genes under high temperatures, drought, and ABA stress. Their results showed that 70 *Lhc* genes responded to high temperatures, drought, and ABA stress. Zhang et al. [59] analyzed the response of *BnLhcbs* to cold stress in *Brassica napus* and showed that overexpression of the *BnLhcb3.4* gene significantly enhanced frost resistance in transgenic *Arabidopsis thaliana* and increased ABA sensitivity. In this study, in the hub genes analyzed using WGCNA, four genes encoded Lhc proteins, namely *BraA09g003480.3.5C*, *BraA09g047590.3.5C*, *BraA02g039380.3.5C*, and *BraA10g012700.3.5C*. They were significantly upregulated after calcium deficiency treatment in the resistant material, but there was no differential expression in the susceptible material. Therefore, we speculated that these four *Lhc* genes play roles in tipburn resistance in Chinese cabbage by regulating photosynthesis.

A common feature of plant responses to stress is reactive oxygen species (ROS) production, which changes the redox homeostasis of cells and results in oxidative stress [60,61]. As part of the stress response, plants produce glutathione (GSH). GSH plays an antioxidant role by scavenging ROS and participates in the ascorbic acid–glutathione cycle to eliminate damaging peroxides [62]. In this study, hub gene *BraA03g058010.3.5C*, analyzed using WGCNA, encoded glutathione peroxidase, which can catalyze GSH into GSSG and promote the decomposition of H_2_O_2_, thereby protecting the structure and function of the cell membrane from the interference and damage of peroxides. This study has shown that the Arabidopsis homologous gene *AT4G31870* was involved in the establishment of photooxidation stress tolerance and the basic resistance to *Pseudomonas syringae* infection [63]. *BraA03g058010.3.5C* may also be involved in tipburn resistance in Chinese cabbage. This analysis showed that the 13 hub genes analyzed using WGCNA may play a key role in tipburn resistance in Chinese cabbage.

## 4. Materials and Methods

### 4.1. Plant Materials and Culture Conditions

In this study, Y578-2 (tipburn-resistant material) and Y920-2 (tipburn-susceptible material) were selected and provided by the Leafy Vegetable Research Group, Institute of Vegetable, Henan Academy of Agricultural Sciences (Zhengzhou, China). After germination, the seedlings were cultured in Hoagland nutrient solution (Supplementary Table S6) and identified using the hydroponic method. The culture conditions were as follows: temperature of 25/22 °C, light–dark cycle of 16/8 h and illumination level of 100 mol/(m^2^×s). Plants at the three-leaf one-heart stage with the same growth vigor were selected from the two lines and treated with different calcium concentrations: normal calcium (C100) and calcium deficiency (C0). The leaves were then collected at 1, 10, and 26 days after calcium treatment for transcriptome sequencing. They were divided into 12 groups, with 3 replicates in each group and a total of 36 samples. The sample numbers are shown in Table 2.

### 4.2. Determination of Antioxidant Enzymes Activities

SOD, POD, and CAT activity in leaves and root activity were measured in response to different treatments using assay kits (Geruisi, Suzhou, China). The root activity kit provides a simple, sensitive and rapid determination method. Modified nitrogen tetrazolium salt is used as a hydrogen receptor, and the colored formazan substance is easily soluble in water. The dehydrogenase activity was determined by measuring the absorbance at 460 nm.

### 4.3. Library Construction and Sequencing

Total RNA was extracted from the leaves of two materials, Y578-2 and Y920-2. The RNA purity and concentration were detected using a NanoDrop 2000 spectrophotometer (Thermo Fisher Scientific, Wilmington, DE, USA). RNA integrity was determined using Agient2100/LabChip GX (Agilent Technologies, Santa Clara, CA, USA). After the samples were qualified, the library was constructed. Eukaryotic mRNA was enriched by magnetic beads with Oligo (dT), and fragmentation buffer was added to randomly interrupt mRNA. The first cDNA chain and the second chain were synthesized with mRNA as the template, and the cDNA was purified. The purified double-stranded cDNA was subjected to terminal repair and A-tail addition, and the sequencing adapter was connected. The fragment size was selected with AMPure XP beads. The cDNA library was obtained using PCR. After library quality inspection, PE150 mode sequencing was performed using the Illumina NovaSeq6000 sequencing platform (San Diego, CA, USA).

### 4.4. Differential Expression Analysis and Gene Annotation

By filtering the raw data and checking the sequencing error rate and GC content distribution, clean reads were obtained for subsequent analysis. HISAT2 software was used to align the Chinese cabbage genome (http://www.brassicadb.cn/ (accessed on 6 February 2025)) and to obtain mapped data [64].

The number and transcript length of mapped reads in the samples were normalized and standardized by FPKM as an indicator of transcript or gene expression level [65].

DESeq2 software was used to calculate the expression of 3 biological replicates, and |log2FC| ≥ 1 and FDR < 0.05 were used as screening criteria for DEGs. The identified DEGs were compared using the Non-Redundant Protein Sequence, Nucleotide Sequence, Uniprot, Clusters of Orthologous Groups, Pfam, Gene Ontology, and KEGG databases to obtain gene annotation information [66].

### 4.5. Weighted Gene Co-Expression Network Analysis

To understand the gene association patterns among samples, the WGCNA package in R was used to analyze the DEGs in two Chinese cabbage varieties during different periods and under different treatments, and a co-expression network was constructed [67]. Cluster analysis was performed based on the gene expression changes. Each line represented a gene, and similar genes were clustered into a branch to form a cluster tree. Different branches represented different gene modules, each of which was composed of a group of highly interrelated genes with similar expression changes associated with specific physiological processes. According to the correlation between the gene module and the sample phenotype, the genes of the key module were determined for subsequent analysis. Cytoscape 3.8.0 software was used to visualize the interaction network of the hub genes in the module.

### 4.6. qRT-PCR Validation

Several DEGs were randomly selected, and qRT-PCR experiments were conducted to verify whether the expression trend of DEGs was consistent with the transcriptome data. Total RNA was extracted using a plant RNA extraction kit (TaKaRa, Beijing, China), and cDNA was synthesized as a template using MonScript^TM^ RTIII All-in-One Mix with a dsDNase Kit from Monad Biotechnology Company (Wuhan, China). *GAPDH* was selected as the reference gene for Chinese cabbage: GACTGGAGAGGTGGAAGAGCC, ACTGAAACATCAACGGTGGGA. The 9 pairs of qRT-PCR primers (Supplementary Table S4) used in this study were designed with DNAMAN Version 9 software and synthesized by Sangon Biotech Co., Ltd. (Shanghai, China). We combined the following reagents: 5.0 μL of 2× TB Green Premix Ex Taq, 0.8 µL of the forward primer, 0.8 µL of the reverse primer, 1.2 µL of cDNA, and 2.2 µL of ddH_2_O. qRT-PCR was performed using a Roche Light Cycler 480II real-time fluorescence quantitative PCR instrument (Roche Applied Sciences, Beijing, China) with the following procedure: 95 °C for 30 s; 45 cycles of 95 °C for 5 s, 60 °C for 20 s, and 72 °C for 30 s; 95 °C for 5 s; 60 °C for 1 min; and 50 °C for 30 s. Three biological replicates were set for each sample, and the relative gene expression level was calculated using the 2^−ΔΔCT^ method [68].

## 5. Conclusions

In this study, we performed transcriptome analysis of two Chinese cabbage DH lines, both tipburn-resistant and tipburn-susceptible, to explore candidate genes involved in the regulation mechanism of tipburn resistance. Combined with phenotypic observation and physiological index determination results, 10 days after calcium deficiency treatment was the key period for differences in resistance between resistant and susceptible materials. WGCNA was used to analyze the correlation between the gene expression patterns in resistant and susceptible materials and tipburn resistance at different stages after treatment with different calcium concentrations. Three gene modules closely related to tipburn resistance were obtained. Co-expression regulation analysis was performed with the genes in these modules, and the following 13 hub genes were identified: *BraA02g039380.3.5C*, *BraA03g028390.3.5C*, *BraA03g038590.3.5C*, *BraA03g058010.3.5C*, *BraA05g008420.3.5C*, *BraA06g002610.3.5C*, *BraA07g024120.3.5C*, *BraA07g029300.3.5C*, *BraA09g003480.3.5C*, *BraA09g047590.3.5C*, *BraA09g047600.3.5C*, *BraA10g012700.3.5C*, and *BraA10g017110.3.5C*. These genes may be involved in the disease resistance of tipburn by regulating the absorption and transport of calcium ions, cell wall metabolism and photosynthesis.

## Figures and Tables

**Table 1:** Annotation information of 13 hub genes.

Gene ID	Location	*A. thaliana*	Gene Function
*BraA02g039380.3.5C*	26,487,248–26,488,449 (A02)	*AT3G27690* (LHCB2)	It encodes the light-harvesting chlorophyll a/b-binding (Lhc) proteins.
*BraA03g028390.3.5C*	14,016,000–14,017,835 (A03)	*AT4G04020* (FIB)	It is regulated by abscisic acid response regulators. It is involved in abscisic acid-mediated photoprotection.
*BraA03g038590.3.5C*	18,860,995–18,862,826 (A03)	*AT3G17800*	It encodes a plant G-box-binding factor.
*BraA03g058010.3.5C*	29,927,476–29,929,090 (A03)	*AT4G31870* (GPX7)	It encodes glutathione peroxidase.
*BraA05g008420.3.5C*	4,412,814–4,414,921 (A05)	*AT2G36870* (XTH32)	It encodes a xyloglucan endotransglycosylase.
*BraA06g002610.3.5C*	1,539,146–1,540,183 (A06)	*AT1G51620*	It encodes LRR receptor-like serine/threonine-protein kinase (LRR-RLK).
*BraA07g024120.3.5C*	18,613,864–18,614,418 (A07)	*AT1G56170* (NF-YC2)	It encodes a nuclear transcription factor Y (NF-Y).
*BraA07g029300.3.5C*	21,156,877–21,158,706 (A07)	*AT1G74420* (FUT3)	It encodes xyloglucan fucosyltransferase, based on similarity to FUT1, but not functionally redundant with FUT1.
*BraA09g003480.3.5C*	2,050,532–2,051,720 (A09)	*AT3G27690* (LHCB2)	It encodes the light-harvesting chlorophyll a/b-binding (Lhc) proteins that constitute the antenna system of the photosynthetic apparatus.
*BraA09g047590.3.5C*	34,898,886–34,900,595 (A09)	*AT3G54890* (LHCA1)	It encodes the light-harvesting chlorophyll a/b-binding (Lhc) proteins.
*BraA09g047600.3.5C*	34,901,365–34,902,146 (A09)	*AT3G54900* (GRXS14)	It encodes glutaredoxin-S14/CAX1 interaction protein. It activates the *CAX1* gene calcium transport activity and plays a role in plant signal transduction and response to stress.
*BraA10g012700.3.5C*	10,248,439–10,249,537 (A10)	*AT5G54270* (LHCB3)	It encodes the light-harvesting chlorophyll a/b-binding (Lhc) proteins.
*BraA10g017110.3.5C*	12,924,138–12,926,370 (A10)	*AT5G59220* (PP2C)	It is a negative regulator of osmotic stress and ABA signaling.

**Table 2:** The thirty-six transcriptome sequencing samples.

Material	Sampling Time	Calcium Treatment	Sample Number		
Y578-2 (R)	1 DAT	100Ca	R1C100-1	R1C100-2	R1C100-3
		0Ca	R1C0-1	R1C0-2	R1C0-3
	10 DAT	100Ca	R10C100-1	R10C100-2	R10C100-3
		0Ca	R10C0-1	R10C0-2	R10C0-3
	26 DAT	100Ca	R26C100-1	R26C100-2	R26C100-3
		0Ca	R26C0-1	R26C0-2	R26C0-3
Y920-2 (S)	1 DAT	100Ca	S1C100-1	S1C100-2	S1C100-3
		0Ca	S1C0-1	S1C0-2	S1C0-3
	10 DAT	100Ca	S10C100-1	S10C100-2	S10C100-3
		0Ca	S10C0-1	S10C0-2	S10C0-3
	26 DAT	100Ca	S26C100-1	S26C100-2	S26C100-3
		0Ca	S26C0-1	S26C0-2	S26C0-3

## Data Availability

The original contributions presented in the study are publicly available. The transcriptome data can be found here: China National GeneBank Database (CNGBdb) under accession number CNP0006380.

## Supplementary Materials

The following supporting information can be downloaded at: https://www.mdpi.com/article/10.3390/plants14040527/s1, Figure S1: Principal component analysis between samples; Figure S2: Heat maps showing all genes in black, blue and turquoise module; Figure S3: Heat map of other transcription factor family gene expression; Table S1: Transcriptome sequencing data output; Table S2: All genes identified in the samples; Table S3: Differentially expressed genes in each of the different comparison groups; Table S4: Primers used for qRT-PCR in this study; Table S5: One-way ANOVA test on the transcriptome FPKM values and qPCR data; Table S6: Hoagland nutrient solution element formula.
