# Supplementary material for: Transcriptome Analysis Revealed Hub Genes Related to Tipburn Resistance in Chinese Cabbage (Brassica rapa L. ssp. pekinensis)
Source: Plants (Basel). 2025 Feb 9;14(4):527. doi: 10.3390/plants14040527 (PMC11859387; doi:10.3390/plants14040527)
Supplement: Supplementary file 1 [file plants-14-00527-s001.zip › Supplemental Table S5.pdf]

For each gene, we performed respectively one-way ANOVA test on the transcriptome FPKM values and qPCR data of the two materials.

BraA01g000700.3.5C

	1day-100Ca	1day-0Ca	10day-100Ca	10day-0Ca	26day-100Ca	26day-0Ca
Y578-2 (RNA-seq)	2.51±0.56f	10.06±0.94e	32.27±1.16d	160.37±4.94a	60.42±1.04c	105.22±0.90b
Y578-2 (qRT-PCR)	1.01±0.07f	3.55±0.74e	6.26±0.07d	43.95±2.14a	11.07±0.61c	24.4±1.66b
Y920-2 (RNA-seq)	2.35±0.73d	1.86±0.11d	4.83±0.98c	12.88±0.50a	4.87±0.72c	6.75±0.33b
Y920-2 (qRT-PCR)	0.99±0.06d	1.09±0.06d	1.79±0.03c	4.27±0.20a	1.77±0.02c	2.13±0.16b

BraA03g046790.3.5C

	1day-100Ca	1day-0Ca	10day-100Ca	10day-0Ca	26day-100Ca	26day-0Ca
Y578-2 (RNA-seq)	82.54±1.54f	202.41±10.01d	166.67±7.83e	231.13±7.07c	272.33±11.71b	378.05±14.99a
Y578-2 (qRT-PCR)	1.01±0.15c	1.76±0.04b	0.30±0.02e	0.62±0.073	0.97±0.16c	1.97±0.06a
Y920-2 (RNA-seq)	5.28±0.64e	6.43±0.92de	7.74±0.843cd	8.63±1.16c	19.68±0.59b	35.91±1.37a
Y920-2 (qRT-PCR)	1.00±0.05cd	0.93±0.03d	0.98±0.02cd	1.04±0.05c	1.52±0.04b	2.31±0.062a

BraA03g047660.3.5C

	1day-100Ca	1day-0Ca	10day-100Ca	10day-0Ca	26day-100Ca	26day-0Ca
Y578-2 (RNA-seq)	3.66±0.80e	60.54±2.35b	6.12±0.18e	25.34±3.26c	14.00±1.09d	87.82±7.10a
Y578-2 (qRT-PCR)	1.02±0.24d	13.29±2.22b	0.74±0.05d	4.91±0.10c	1.66±0.07d	21.41±0.61a
Y920-2 (RNA-seq)	0.09±0.12bc	0.10±0.14bc	0.00±0.00c	0.27±0.04a	0.2±0.03ab	0.18±0.06ab
Y920-2 (qRT-PCR)	1.01±0.08c	2.14±0.19c	1.28±0.45c	8.93±0.65a	4.29±1.13b	2.44±1.57bc

BraA03g058010.3.5C

	1day-100Ca	1day-0Ca	10day-100Ca	10day-0Ca	26day-100Ca	26day-0Ca
Y578-2 (RNA-seq)	14.10±0.28f	29.72±1.49e	38.58±3.02d	90.65±1.22b	67.86±1.19c	109.11±7.34a
Y578-2 (qRT-PCR)	1.01±0.18d	1.22±0.03d	1.57±0.07d	4.61±0.22b	3.28±0.67c	6.78±0.70a
Y920-2 (RNA-seq)	7.37±0.87d	9.29±1.59d	14.74±1.37c	20.01±3.72b	24.53±0.87a	15.57±0.19c
Y920-2 (qRT-PCR)	1.01±0.14c	1.27±0.16bc	1.34±0.06bc	2.00±0.24a	2.23±0.26a	1.51±0.19b

BraA06g038740.3.5C

	1day-100Ca	1day-0Ca	10day-100Ca	10day-0Ca	26day-100Ca	26day-0Ca
Y578-2 (RNA-seq)	20.14±0.90d	107.16±6.35b	66.09±2.84c	152.27±3.35a	14.19±2.32d	73.87±9.95c
Y578-2 (qRT-PCR)	1.04±0.33c	6.57±0.52a	1.71±0.08c	6.99±0.65a	1.11±0.20c	3.83±0.50b
Y920-2 (RNA-seq)	2.54±0.66c	2.30±0.67c	5.83±0.94b	5.62±1.35b	1.68±0.42c	19.6±0.30a
Y920-2 (qRT-PCR)	1.00±0.01c	0.75±0.01e	1.28±0.02b	0.91±0.06d	0.39±0.03f	3.10±0.03a

BraA07g043670.3.5C

	1day-100Ca	1day-0Ca	10day-100Ca	10day-0Ca	26day-100Ca	26day-0Ca
Y578-2 (RNA-seq)	0.88±0.19c	3.72±0.69a	0.39±0.13c	0.95±0.30bc	1.82±0.12b	11.00±0.71a
Y578-2 (qRT-PCR)	0.99±0.02d	1.51±0.05b	0.12±0.04e	0.93±0.03d	1.19±0.04c	3.35±0.05a
Y920-2 (RNA-seq)	1.43±0.42b	0.95±0.09c	0.12±0.07d	0.40±0.18d	1.85±0.04a	0.11±0.03d
Y920-2 (qRT-PCR)	1.00±0.12b	0.48±0.05c	0.06±0.01d	0.11±0.03d	1.14±0.10a	0.03±0.01d

BraA08g033390.3.5C

	1day-100Ca	1day-0Ca	10day-100Ca	10day-0Ca	26day-100Ca	26day-0Ca
Y578-2 (RNA-seq)	16.69±2.18d	51.89±3.82a	26.55±1.74c	31.84±0.67b	54.97±2.06a	52.65±1.37a
Y578-2 (qRT-PCR)	1.00±0.03d	2.41±0.04b	1.02±0.06d	1.55±0.12c	2.87±0.02a	2.81±0.10a
Y920-2 (RNA-seq)	8.00±0.83b	7.32±0.60b	12.50±0.43a	11.47±2.08a	12.21±0.49a	12.75±0.74a
Y920-2 (qRT-PCR)	1.00±0.09b	1.00±0.06b	1.17±0.02a	1.14±0.02a	1.16±0.02a	1.17±0.03a

BraA09g047600.3.5C

	1day-100Ca	1day-0Ca	10day-100Ca	10day-0Ca	26day-100Ca	26day-0Ca
Y578-2 (RNA-seq)	32.41±3.05d	70.06±2.73a	40.03±2.06c	48.82±1.64b	18.43±2.25e	41.62±3.28c
Y578-2 (qRT-PCR)	1.00±0.11d	3.87±0.20a	1.23±0.02c	1.42±0.02c	0.83±0.06d	1.85±0.03b
Y920-2 (RNA-seq)	2.01±0.19b	1.68±0.12b	4.89±0.78a	3.80±1.47a	4.45±0.81a	5.37±0.78a
Y920-2 (qRT-PCR)	1.01±0.18b	1.04±0.07b	1.50±0.10a	1.52±0.02a	1.49±0.01a	1.52±0.02a

BraA10g023710.3.5C

	1day-100Ca	1day-0Ca	10day-100Ca	10day-0Ca	26day-100Ca	26day-0Ca
Y578-2 (RNA-seq)	15.50±1.74d	22.76±1.30bc	24.55±0.59b	28.77±1.42a	8.75±1.40e	22.00±0.14c
Y578-2 (qRT-PCR)	1.00±0.07d	1.52±0.10b	1.39±0.01c	1.63±0.01a	0.44±0.01e	1.39±0.03c
Y920-2 (RNA-seq)	1.60±0.64a	1.21±0.12a	1.34±0.22a	1.16±0.12a	0.98±0.26a	0.98±0.21a
Y920-2 (qRT-PCR)	1.00±0.07a	0.90±0.01b	0.91±0.06ab	0.91±0.03ab	0.80±0.03c	0.88±0.06bc
