# Supplementary material for: Transcriptome Analysis Revealed Hub Genes Related to Tipburn Resistance in Chinese Cabbage (Brassica rapa L. ssp. pekinensis)
Source: Plants (Basel). 2025 Feb 9;14(4):527. doi: 10.3390/plants14040527 (PMC11859387; doi:10.3390/plants14040527)
Supplement: Supplementary file 1 [file plants-14-00527-s001.zip › Supplemental Table S6.pdf]

Supplemental Table S6. Hoagland nutrient solution element formula.

Reagent	Molecular weight (g/mol)	Concentration (mmol/L)	Mass concentration (mg/L)	Mother liquor multiple
$Ca(NO_3)_2 \cdot 4H_2O$	236.14	4.0018	94.50	100x
KNO_3	101.10	6.0039	60.70	100x
$NH_4H_2PO_3$	115.02	0.9998	11.50	100x
$MgSO_4 \cdot 7H_2O$	246.47	0.9778	24.10	100x
$FeSO_4 \cdot 7H_2O$	278.02	0.5999	8.34	500x
$EDTA\text{-}Na_2$	372.24	0.0271	5.04	500x
H_3BO_3	61.83	0.0463	1.43	500x
KI	166.00	0.0050	0.42	500x
$MnSO_4 \cdot H_2O$	169.02	0.0096	0.81	500x
$ZnSO_4 \cdot 7H_2O$	287.56	0.0007	0.11	500x
$CuSO_4 \cdot 5H_2O$	249.69	0.0003	0.04	500x
$CoCl_2 \cdot 6H_2O$	237.93	0.0001	0.01	500x
$(NH_4)_6Mo_7O_{24} \cdot 4H_2O$	1235.86	0.0002	0.01	500x
