# Supplementary figures and images for: Transcriptome Analysis Revealed Hub Genes Related to Tipburn Resistance in Chinese Cabbage (Brassica rapa L. ssp. pekinensis)
Source: Plants (Basel). 2025 Feb 9;14(4):527. doi: 10.3390/plants14040527 (PMC11859387; doi:10.3390/plants14040527)

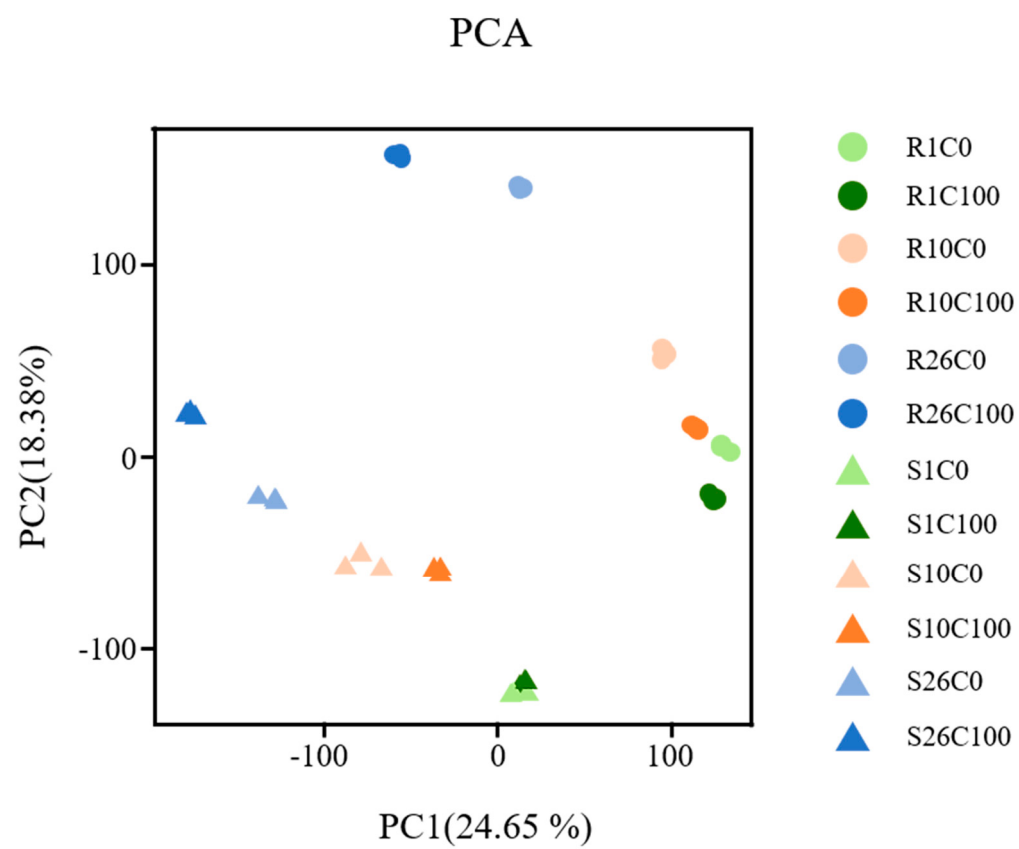

Figure S1

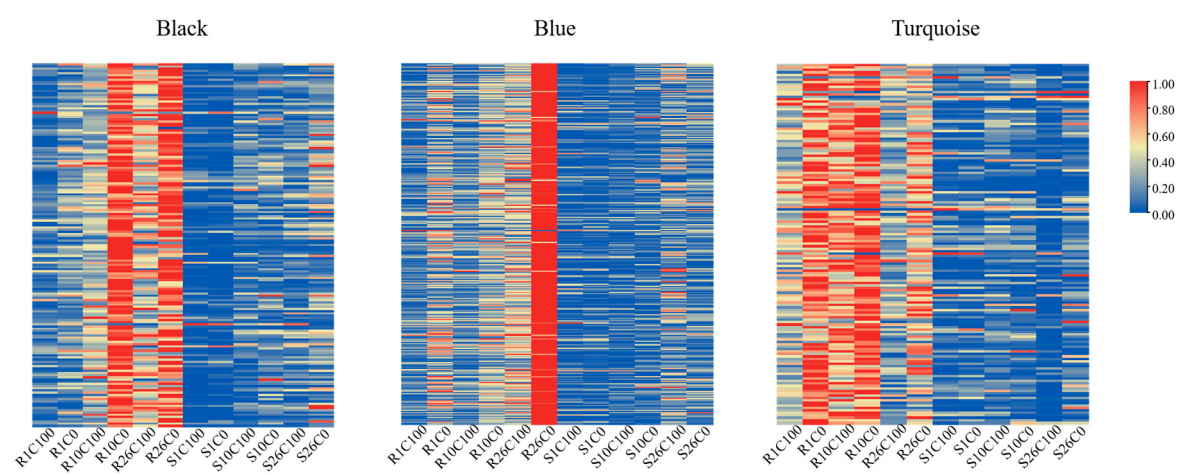

**Figure S2**

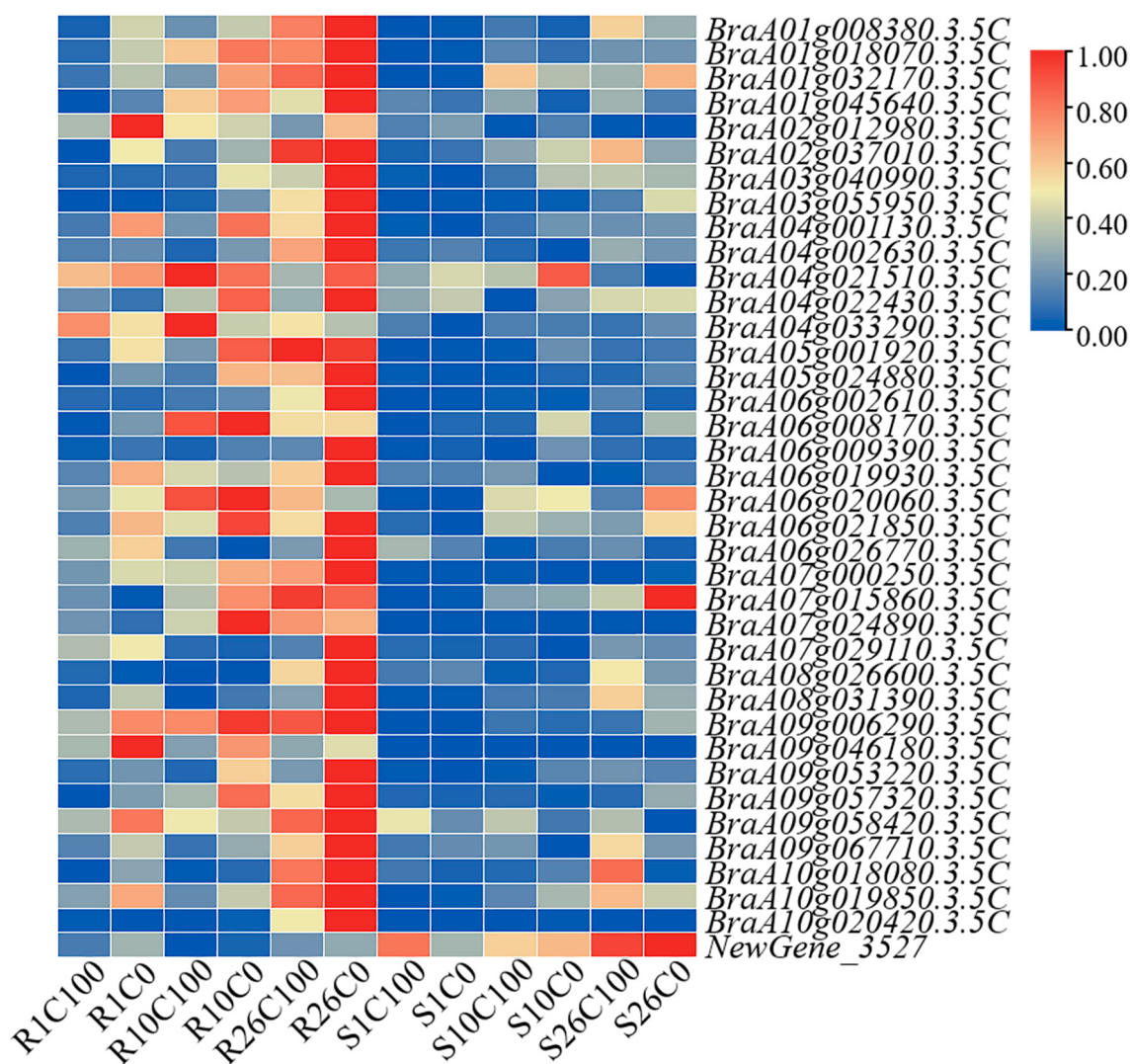

Figure S3

Supplement: Supplementary file 1 [file plants-14-00527-s001.zip › Supplemental Figures S1-S3.pdf]
